# Pleiotropic Associations of *RARRES2* Gene Variants and Circulating Chemerin Levels: Potential Roles of Chemerin Involved in the Metabolic and Inflammation-Related Diseases

**DOI:** 10.1155/2018/4670521

**Published:** 2018-03-12

**Authors:** Leay-Kiaw Er, Semon Wu, Lung-An Hsu, Ming-Sheng Teng, Yu-Ching Sun, Yu-Lin Ko

**Affiliations:** ^1^The Division of Endocrinology and Metabolism, Department of Internal Medicine, Taipei Tzu Chi Hospital, Buddhist Tzu Chi Medical Foundation, New Taipei City, Taiwan; ^2^School of Medicine, Tzu Chi University, Hualien, Taiwan; ^3^Department of Research, Taipei Tzu Chi Hospital, Buddhist Tzu Chi Medical Foundation, New Taipei City, Taiwan; ^4^Department of Life Science, Chinese Culture University, Taipei, Taiwan; ^5^The First Cardiovascular Division, Department of Internal Medicine, Chang Gung Memorial Hospital and Chang Gung University College of Medicine, Taoyuan, Taiwan; ^6^Department of Laboratory Medicine, Chang Gung Memorial Hospital, Taipei, Taiwan; ^7^The Division of Cardiology, Department of Internal Medicine and Cardiovascular Center, Taipei Tzu Chi Hospital, Buddhist Tzu Chi Medical Foundation, New Taipei City, Taiwan

## Abstract

Chemerin, an adipokine and inflammatory mediator, is associated with metabolic, inflammation- and immune-mediated diseases. The genetic, clinical, and biomarker correlates of circulating chemerin levels have not been completely elucidated. We analyzed the determinants and correlates of retinoic acid receptor responder 2 (*RARRES2*; encoding chemerin) gene variants and chemerin levels in the Taiwanese population. In total, 612 individuals were recruited. Clinical and metabolic phenotypes, 13 inflammatory markers, 5 adipokines, and 6 single-nucleotide polymorphisms (SNPs) covering the *RARRES2* region were analyzed. High chemerin levels and chemerin level tertiles were positively associated with multiple metabolic phenotypes and circulating inflammatory marker and adipokine levels and negatively associated with high-density lipoprotein cholesterol and adiponectin levels and estimated glomerular filtration rates (eGFRs). Genotype and haplotype analyses showed that *RARRES2* SNPs were significantly associated with chemerin, fibrinogen, interleukin 6, and lipocalin 2 levels. Stepwise logistic regression analysis showed that C-reactive protein level, leptin level, triglyceride level, eGFR, rs3735167 genotypes, sex, and soluble P-selectin level were independently associated with chemerin levels. In conclusion, pleiotropic associations were noted between *RARRES2* variants, circulating chemerin levels and multiple metabolic phenotypes and inflammatory marker levels. This study provides further evidence for the potential roles of chemerin in metabolic and inflammation-related diseases.

## 1. Introduction

Chemerin, an adipokine and a chemoattractant protein [1–3], is mainly expressed in white adipose tissues [2, 4]; it is induced during adipocyte differentiation with elevated circulating levels in obesity [5]. In addition to adipose tissue, chemerin is expressed in other cell types involved in innate and adaptive immunity, which promotes the recruitment of immune cells to the site of injury [3]. Chemerin is secreted in an inactive form as prochemerin and is processed by various proteases involved in coagulation to produce active and inactive forms of chemerin [6]. Upon proteolytic activation by proteases, different chemerin fragments with pro- or anti-inflammatory action can be produced, depending on the class of proteases in the microenvironment. Previous studies demonstrated the pleiotropic role of chemerin in diverse biological processes including immune response regulation [3], inflammation [7–9], glucose metabolism [10], and angiogenesis [11, 12].

Studies have suggested that chemerin is a predictor of metabolic syndrome [7, 10]. The elevation of chemerin levels is positively correlated with detrimental effects on glucose, lipid, and cytokine homeostasis and may act as a link among obesity, inflammation, and other metabolic derangement [13]. Plasma chemerin is increased in chronic inflammatory diseases, and levels of chemerin are correlated with those of proinflammatory cytokines such as tumor necrosis factor, interleukin (IL) 6, and C-reactive protein (CRP) [7–9]. Furthermore, chemerin is involved in the pathophysiology of rheumatoid arthritis [14], ulcerative colitis, Crohn's disease [15], psoriasis, chronic pancreatitis [16], chronic renal disease [17], and liver disease [18].

Chemerin binds to three receptors including CMKLR1 (also known as ChemR23), chemokine receptor-like 2 (CCRL2), and G protein-coupled receptor 1 (GPR1); and the chemerin–CMKLR1 axis plays multiple roles in the control of inflammation, metabolism, and carcinogenesis in different organs and systems [19]. CMKLR1 is also present on the endothelium of blood vessels as well as on underlying smooth muscle cell layers [20]. CMKLR1 agonism leads to changes in vascular tone, causing hypertension; the damaged endothelium uncovers CMKLR1 on smooth muscle cells, thus promoting the development of atherosclerosis. In addition, a positive and independent correlation has been observed between circulating levels of chemerin and risks of coronary artery disease (CAD), ischemic stroke, and carotid plaque instability [21, 22].

Serum chemerin levels have been shown to be moderately heritable, with 16%–25% of variations attributed to genetic factors [11, 23]. Variants of retinoic acid receptor responder 2 (*RARRES2*), the gene encoding chemerin, have been demonstrated to be associated with increased chemerin levels, visceral fat mass in nonobese individuals, and increased incidence of metabolic syndrome [11, 23–26]. Coexpression network analyses of gluteal and abdominal adipose tissue revealed that rs10282458 in the *RARRES2/REPIN1* region modulated *RARRES2* expression and were associated with body mass index (BMI) [25]. To our knowledge, there are no previous data about the association between the levels of chemerin and the inflammatory markers SAA, MMP1, MMP2, and MMP9 and the adipokines lipocalin 2 and GDF15 in human. Furthermore, literature about the correlation of *RARRES2* variants and various inflammatory markers and adipokines is also lacking. Haplotypes present a record of evolutionary history more accurately than do individual SNPs and capture the LD patterns of a genomic region more adequately. Susceptibility genes in complex diseases such as metabolic and inflammation-related diseases may be identified by haplotypes more effectively than by individual SNPs. The present study was conducted to analyze the association of genetic, clinical, and biomarker predictors with circulating chemerin levels, as well as the association of *RARRES2* genotypes and haplotypes with various clinical phenotypes and biomarker levels in the Taiwanese population.

## 2. Participants and Methods

### 2.1. Participants

Han Chinese participants were enrolled during routine cardiovascular health examinations between October 2003 and September 2005 at Chang Gung Memorial Hospital, Taoyuan, Taiwan. Responses to a questionnaire on medical history and lifestyle characteristics were recorded. The participants underwent a physical examination, with the body height and weight, waist and hip circumferences, and blood pressure (BP) measured. The participants' BP was measured after 15 minutes of rest in the sitting position. All participants provided written informed consent. Exclusion criteria included having a history of myocardial infarction, stroke, transient ischemic attack, cancer, or current renal or liver disease. Furthermore, five participants younger than 18 years were excluded from the analysis. Thus, a total of 612 participants, age ranged between 19 and 82 years (323 men [mean age: 45.6 ± 10.0 years] and 289 women [mean age: 47.0 ± 10.0 years]), were included in the analysis. According to the Asian criteria of the 2004 World Health Organization Expert Consultation, overweight and obesity were defined as BMI ≥ 25 kg/m^2^ [27]. Current smokers were defined as those who regularly smoked cigarettes at the time of the survey. The Ethics Committee of Taipei Tzu Chi Hospital, Buddhist Tzu Chi Medical Foundation, approved this study (IRB number: 04-XD01-001). Diabetes mellitus was defined according to the guidelines of the American Diabetes Association as a fasting plasma glucose level of ≥126 mg/dL or a history of receiving hypoglycemic agents. Moreover, hypertension was defined as a systolic BP of ≥140 mmHg and/or a diastolic BP of ≥90 mmHg, or a history of receiving antihypertensive drugs. Metabolic syndrome was defined according to the Adult Treatment Panel III Asian criteria.  Table 1 presents the baseline characteristics of the study participants according to the tertiles of circulating chemerin levels.

### 2.2. Genomic DNA Extraction and Genotyping

Genomic DNA was extracted as previously reported [28]. Oligonucleotide primers were generated to amplify genomic DNA fragments containing single-nucleotide polymorphisms (SNPs), as reported in the National Center for Biotechnology Information SNP database (http://www.ncbi.nlm.nih.gov/SNP). On the basis of the HapMap database, we analyzed six SNPs with selected tagSNPs by running the tagger program implemented in SNPinfo Web Server and with the coverage of the *RARRES2* region (encoding chemerin; linkage disequilibrium [LD] threshold = 0.8 and minor allele frequency ≥ 0.2). Genotyping for the studied polymorphisms was performed using TaqMan SNP genotyping assays obtained from Applied Biosystems (ABI, Foster City, CA, USA).

### 2.3. Laboratory Examinations

Venous blood was collected in the morning after an overnight fast, and serum and plasma samples were obtained through centrifugation at 3000 ×g for 15 minutes at 4°C and then stored at −80°C prior to analyses. The following variables were performed according to the methods described by Hsu et al. [29]. Glucose levels were enzymatically determined using the hexokinase method, and total cholesterol (TC) and triglyceride (TG) levels were measured through automatic enzymatic colorimetry. High-density lipoprotein cholesterol (HDL-C) levels were enzymatically measured after phosphotungsten/magnesium precipitation. LDL-C levels were calculated using the Friedewald formula; however, in patients with a TG level > 400 mg/dL, low-density lipoprotein cholesterol (LDL-C) levels were measured using commercial reagents with a standard protocol. Serum insulin levels were measured using an immunoradiometric assay (BioSource, Nivelles, Belgium). The homeostasis model assessment of insulin resistance (HOMA-IR) index was calculated using the following formula: HOMA-IR = fasting serum insulin (*μ*U/mL) × fasting plasma glucose (mmol/L)/22.5. Estimated glomerular filtration rate (eGFR) was calculated as previously reported [30]. Circulating plasma levels of chemerin, growth differentiation factor (GDF) 15, matrix metalloproteinase (MMP) 1, soluble P-selectin (sP-selectin), and soluble tumor necrosis factor receptor (sTNFR) II, as well as serum levels of lipocalin (LCN) 2, MMP2, and resistin, were measured using commercially available enzyme-linked immunosorbent assay (ELISA) kits (R&D, Minneapolis, MN, USA). Other markers, namely, serum CRP, serum amyloid A (SAA), soluble intercellular adhesion molecule 1 (sICAM1), soluble vascular cell adhesion molecule 1 (sVCAM1), soluble E-selectin (sE-selectin), adiponectin, leptin, MMP9, and plasma monocyte chemotactic protein 1 were measured using an in-house sandwich ELISA kit. All in-house kits showed good correlation levels compared with those of commercially available ELISA kits (Supplementary References available here). Furthermore, serum insulin levels were measured using an immunoradiometric assay (BioSource, Nivelles, Belgium). Plasma fibrinogen levels were determined using the Clauss method adapted for a Sysmex CA1-1500 instrument (Kobe, Japan). ELISA and genotyping were performed by laboratory personnel blinded to the clinical status of the participants. Overall, the intra- and interassay variability of coefficients were within the range of 1.8% to 10.9% (Supplementary Table 1).

### 2.4. Statistical Analysis

The chi-squared or chi-squared test for trend was used to determine differences in the categorical data distribution. The clinical characteristics of the continuous variables are expressed as means ± standard deviation and were tested using a two-sample *t*-test or analysis of variance, except when the distribution was strongly skewed, in which case the median and interquartile ranges are provided. Pearson and partial correlation coefficients were used to analyze the relationship between chemerin levels and clinical and biochemical factors. A generalized linear model was used to analyze chemerin levels associated with the predictors of the investigated genotypes and confounders. Linear regression analysis with the stepwise method was also performed to determine the independent correlates of chemerin levels. All biomarker levels were logarithmically transformed before statistical analysis to adhere to a normality assumption. The Bonferroni correction method was used to address the accumulated errors from multiple testing by determining the corrected cutoff for each *P* value with the total numbers of tests (*n*). The adjusted *P* values < 0.05 were taken as statistically significant. The analysis of deviation from the Hardy–Weinberg equilibrium, estimation of the LD between polymorphisms (Supplementary Figure 1) and haplotypes, was performed using Golden Helix SVS Win32 7.3.1 software.

## 3. Results

### 3.1. Associations between Chemerin Levels and Clinical Correlates

Tables 1, 2, and 3 present the associations between chemerin levels and clinical factors. Even after Bonferroni correction with an adjustment of 17 clinical correlates, participants with higher chemerin levels were found to be significantly older, in addition to having significantly higher BMI values; waist circumference values; systolic, mean, and diastolic BP levels; triglyceride, creatinine, and insulin levels; and HOMA-IR index values; and significantly lower serum HDL-C levels and eGFR in tertile and/or continuous variable analyses. The frequencies of smoking, obesity, hypertension, IR, and metabolic syndrome were also higher with a higher tertile of chemerin levels. Furthermore, we analyzed the associations between chemerin levels and several risk factors for cardiovascular disease. Plasma levels of chemerin were significantly higher in women, current smokers, and participants with hypertension, obesity, IR, and metabolic syndrome (*P* = 0.044, 0.009, 0.015, 6.52 × 10^−9^, 0.001, and 0.004, resp.; Table 4). However, after Bonferroni correction, significant associations were found only in obesity, IR, and metabolic syndrome (*P* = 4.55 × 10^−8^, 0.007, and 0.021, resp.; Table 3).

### 3.2. Associations between Chemerin Levels and Biomarker Correlates

**Figure 1:**
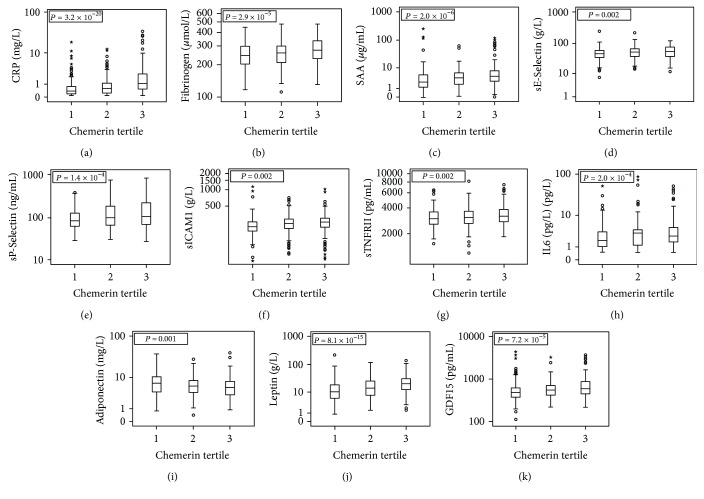
Box and whisker plots analysis for the circulating inflammatory marker and adipokine levels according to chemerin level tertiles. Our data revealed significant associations of multiple inflammatory marker and adipokine levels with chemerin level tertiles (*P* values between 0.002 and 3.2 × 10^−20^). Abbreviations were used as in Table 3. The stars in each plot of the figure revealed participants with biomarker levels over three times the interquartile range (IQR) in each chemerin level tertile. If the value of outlier is between 1.5 and 3 times the IQR, we remark “○.” Note that we have an outlier for CRP in the third tertile of chemerin on truncated coordination of Figure 1.

Tertile and continuous variable analyses revealed that chemerin levels were associated with circulating levels of most of the studied inflammatory markers including CRP, fibrinogen, SAA, sE-selectin, sP-selectin, sICAM1, sTNFRII, MMP1, and MMP2 (all *P* < 0.05). The association was observed even after Bonferroni correction with an adjustment of 13 inflammatory biomarker levels (Figure 1, Table 4, and Supplementary Table 2), considering circulating adipokine levels, leptin, and LCN2 levels were positively, while adiponectin level was negatively, associated with chemerin levels (all *P* < 0.01 after Bonferroni correction).

### 3.3. Associations of the *RARRES2* Locus Genotypes and Haplotypes with Circulating Chemerin Levels

No significant deviation from the Hardy–Weinberg equilibrium was detected for the *RARRES2* locus variants. Strong linkage disequilibrium was observed between SNPs rs7806429 and rs4721 and between SNPs rs3735167 and rs10282458 (Supplementary Figure 1). Variants in or around *RARRES2* were significantly associated with chemerin levels in our Taiwanese cohort (Table 5). After adjustment for clinical covariates, chemerin levels were observed to be significantly associated with the studied polymorphisms rs7806429, rs4721, rs3735167, and rs10282458 using an additive inheritance model (*P* = 0.001, 0.002, 5.27 × 10^−6^, and 1.08 × 10^−5^, resp.) and a dominant model (*P* = 0.009, 0.019, 4.33 × 10^−5^, and 8.91 × 10^−5^, resp.). Moreover, haplotype analysis revealed that *RARRES2* SNPs were significantly associated with chemerin levels (*P* = 0.008 and 0.0002 for haplotypes TTTCTG and CGTTTA, resp.; Table 6).

### 3.4. Associations of the *RARRES2* Locus Genotypes and Haplotypes with Clinical and Biochemical Correlates

Genotype and haplotype analyses revealed that *RARRES2* SNPs were significantly associated with fibrinogen, IL6, and LCN2 levels (maximum *P* = 0.003, 0.007, and 0.008, resp., for rs3735167 and rs10282458 genotypes; and maximum *P* = 0.007, 0.016, and 0.011, resp., for the CGTTTA haplotype in Table 6). None of the *RARRES2* locus genotypes were significantly associated with other clinical or biochemical correlates, with or without adjustment for circulating chemerin levels.

### 3.5. Stepwise Regression Analysis of Chemerin Levels with a General Linear Model in the Study Population

Because we observed that multiple genetic, clinical, and biomarker parameters correlated with circulating chemerin levels, stepwise logistic regression analysis was performed, which revealed that CRP, leptin, triglyceride, eGFR, rs3735167-CC genotype, sex, and sP-selectin level were all independently associated with chemerin levels (*P* < 0.001, <0.001, 0.002, <0.001, 0.001, 0.012, and 0.019, resp.; Table 7).

## 4. Discussion

Our data revealed significant associations of the *RARRES2* genotypes and haplotypes with chemerin, fibrinogen, IL6, and LCN2 levels. In addition, chemerin levels were found to be associated with age, sex, renal function, obesity, IR, multiple metabolic phenotypes, and metabolic syndrome as well as many circulating inflammatory marker and adipokine levels. All of these parameters have been previously reported to be associated with the risk or long-term outcome of atherosclerotic cardiovascular disease. These results enable the understanding of genetic, clinical, and biomarker correlates of circulating chemerin levels in the Taiwanese population and provide further evidence for the potential roles of chemerin in metabolic and inflammation-related diseases.

### 4.1. Associations of Chemerin Levels with Clinical Characteristics

Circulating chemerin levels are associated with multiple metabolic phenotypes. Li et al. conducted a meta-analysis of eight studies enrolling 1787 participants for determining the association between serum chemerin levels and clinical indices in obesity and metabolic syndrome [31]. The meta-analysis results regarding metabolic and obesity markers indicated that triglycerides, total cholesterol, CRP, BMI, total body fat percentage, waist circumference, waist–hip ratio, systolic BP, and leptin levels were positively correlated with chemerin levels. Nevertheless, diastolic BP, LDL-C, and HDL-C were not significantly correlated in the described meta-analysis. However, several studies have reported a positive correlation between chemerin levels and both systolic and diastolic BP values, even after adjustment for age and BMI [2] or systolic BP [32], diastolic BP [33], alone. Concordantly, our data revealed an association of high chemerin levels with nearly all metabolic phenotypes related to metabolic syndrome, including high waist circumference, high serum triglyceride levels, high BP, high HOMA-IR index, and low HDL-C levels. The relationship of chemerin and BP may be explained by inflammatory cytokine-dependent upregulation of *CMKLR1* expression that has been discovered on vascular endothelial cells in human [12]. Chemerin was able to induce functional angiogenesis in these cells [12]. Furthermore, another study also found CMKLR1 receptor on endothelium of blood vessel and their underlying smooth muscle layers [20], and these may potentially affect BP. Chemerin played a role in adipogenesis [4]. Adipocytes are responsible for storing excess calories and may lead to adipocyte hypertrophy and hyperplasia and cause increases intracellular lipids, proinflammatory cytokines, and free fatty acids, which are the risk factors involved in the formation of dyslipidemia [34].

Chemerin has been reported to regulate adipocyte differentiation, lipid homeostasis, and insulin sensitivity [2, 35]. Chemerin showed a relatively strong correlation with clamp-derived insulin sensitivity [36]. In our study, high chemerin levels were associated with a higher incidence of IR and metabolic syndrome, and these results are consistent with those of previous studies [2, 7, 31]. Our data are also compatible with those of other studies that have reported significant and independent correlations of chemerin levels with renal function, even after adjustment for other variables [17, 37].

### 4.2. Associations of Chemerin Levels with Inflammatory Biomarker Levels

Several studies have demonstrated the association of circulating chemerin levels with inflammatory markers such as high-sensitivity CRP (hs-CRP), IL6, and tumor necrotic factor *α* [8, 9]. Moreover, one study reported an association of chemerin with hs-CRP and fibrinogen, even after adjustment for waist circumference [38], indicating that this association cannot be explained by the accumulation of adipose tissue alone. Chemerin, a chemoattractant protein, activates the adhesion of macrophages to fibronectin and VCAM1 in a CMKLR1-dependent manner, and chemerin-stimulated adhesion may be crucial in recruiting and retaining macrophages at inflammation sites and in their further activities [3]. Additionally, chemerin interacts with the endothelium by inducing the production of ICAM1 and E-selectin [39] in children, and it also promotes the release of MMP2, MMP9 [12], and MMP7 [40], which may play a role in blood vessel remodeling and growth in *in vitro* experiments. This relationship between chemerin and MMPs has not been investigated in human. In this study, tertile and continuous variable analyses revealed an association of chemerin levels with multiple markers associated with different stages of chronic inflammation. These markers were as follows: a coagulation factor (fibrinogen), cell adhesion molecules (sICAM1, sE-selectin, and sP-selectin), matrix metalloproteinases (MMP1 and MMP2), and the final common pathway of inflammation (CRP, SAA, IL6, and sTNFRII). These results suggest that chemerin may induce extensive inflammatory processes in various inflammatory disorders. To our knowledge, there are no previous data about the association between the levels of chemerin and various inflammatory markers including SAA, MMP1, MMP2, and MMP9 in human. This study is also the first study of East Asian population investigating the association between the levels of chemerin and various inflammatory markers including fibrinogen, sICAM1, sVCAM1, sE-selectin, sP-selectin, MCP1, SAA, MMP1, MMP2, and MMP9.

### 4.3. Associations of Chemerin Levels with Adipokines Levels

Chemerin, a proinflammatory adipokine, modulates chemotaxis and activities of macrophages [2]. Circulating chemerin levels have been reported to be significantly correlated with obesity [2, 41]. Sell et al. [5] and Weigert et al. [7] have reported a positive association between chemerin levels and resistin and leptin levels. Chu et al. [42] revealed that chemerin and adiponectin reciprocally contribute to metabolic syndrome. In a meta-analysis, adiponectin was negatively correlated with chemerin levels [31]. No previous study has been reported focusing on the correlation between the levels of chemerin and other novel adipokines such as lipocalin 2 and GDF15 in human. This study is also the first study of East Asian population investigating the association between the levels of chemerin and adipokines such as leptin, resistin, lipocalin 2, and GDF15. Our data show that chemerin levels were positively correlated with leptin and GDF15 levels, negatively correlated with adiponectin levels, and not significantly associated with resistin levels. The mentioned results may reveal the potential roles of chemerin in metabolic and inflammation-related diseases.

### 4.4. Chemerin in the Context of Previous Publications for the Same Study Population

This investigation is a substudy of an earlier clinical study that was performed between 2003 and 2005. Correlation analyses of several inflammatory biomarkers have been reported since 2009 [43–48]. When compared to other biomarker levels, such as MMP9, sICAM1, YKL-40, SAA, and GDF15 levels, chemerin levels seemed to have stronger associations with multiple metabolic phenotypes and inflammatory marker levels. Further, inverse associations were found between chemerin and adiponectin levels on the metabolic phenotypes and inflammatory marker levels as previously reported [42, 46, 47]. Circulating biomarkers have been widely used for risk prediction of metabolic and immune-related disorders, including atherosclerotic cardiovascular disease [49]. A recent report has shown that chemerin levels were associated with the prognosis of CAD [37]. Further study may be necessary to elucidate the role of chemerin in predicting the risk and prognosis of various metabolic and inflammatory diseases.

### 4.5. Associations of *RARRES2* SNPs with Circulating Chemerin Levels

Genome-wide association studies have reported an association of *RARRES2* loci with circulating chemerin levels [23, 37]. SNPs, both rs7806429 and rs3735167, were found to be associated with *RARRES2* mRNA expression with possible functional effects and had the lowest *P* values in two genome-wide analyses. By contrast, Leiherer et al. reported no association of the SNP rs4721 with serum chemerin levels [37]. Notably, the SNP rs17173608, which was previously found to be associated with multiple metabolic factors and diseases, was not associated with chemerin levels. Our data revealed that the SNPs rs3735167 and rs10282458, which were in nearly complete LD, had the lowest *P* values for the association with chemerin levels. The SNPs rs7806429 and rs4721 had a strong LD and were significantly associated with chemerin levels in a univariate but not multivariate analysis, whereas rs17173608 was not associated with serum chemerin levels in our study. These results suggested ethnic genetic heterogeneity in the association of *RARRES2* SNPs with chemerin levels; each ethnic population may yield distinct data.

### 4.6. Pleiotropic Effects of *RARRES2* SNPs

Studies have reported the pleiotropic effects of *RARRES2* SNPs, including a varying adiposity status, visceral fat mass, metabolic syndrome, and polycystic ovary syndrome [24–26, 50, 51]. The SNP rs17173608, the most commonly reported SNP associated with all the aforementioned phenotypes in non-Asian populations, was not associated with chemerin levels or various clinical characteristics, inflammatory biomarkers, or adipokines in our study population. The SNP rs17173617, which was reported to have a strong LD with rs3735167, was associated with the risk of DM in a Chinese population [52]. Literatures about the correlation of *RARRES2* variants and various inflammatory markers and adipokines shown in the manuscript are unavailable. Our data showed that *RARRES2* variants were associated with circulating chemerin, fibrinogen, IL6, and LCN2 levels, but not with other clinical phenotypes or biomarker levels in the Taiwanese population. These results warrant a large prospective study with a more complete genotyping analysis, which may facilitate a more comprehensive understanding of the pleiotropic effects of *RARRES2* variants.

### 4.7. Limitations

The main limitation of this study is its cross-sectional design. Further, this is a correlation study with no biological experimental evidence to validate the correlations or to state that chemerin induces extensive inflammatory processes in various diseases. Additional functional and prospective studies will enable to elucidate the mechanism and role of *RARRES2* variants and circulating chemerin levels in the long-term outcomes of metabolic and inflammation-related diseases. The reagent in the R&D Systems ELISA kit recognizes not only bioactive chemerin but also inactive prochemerin and large degraded/inactive chemerin protein fragments. Thus, it would be more informative to assess other bioactivity assays (*β*-arrestin signaling, DiscoverX) or ELISA [53].

In conclusion, this study revealed *RARRES2* variants as the genetic determinants of circulating chemerin, fibrinogen, IL6, and LCN2 levels in the Taiwanese population. The associations of chemerin levels with multiple metabolic phenotypes and inflammatory marker levels were also investigated. Based upon our multiple analyses, we provided further and novel evidence about the important role of chemerin and *RARRES2* variants involved in inflammation and metabolic diseases. Future studies are needed to elucidate the role of chemerin in diverse diseases.

## Figures and Tables

**Table 1:** Baseline characteristics of the study subjects according to tertiles of circulating chemerin levels.

	*N*	Total	1st tertile	2nd tertile	3rd tertile	*P* value
Age (years)	612	46.2 ± 10.0	44.6 ± 9.7	46.1 ± 9.7	48.7 ± 9.7	1.9 × 10^−4^
Waist circumference (cm)	612	85.2 ± 9.6	82.2 ± 9.2	85.3 ± 8.8	88.5 ± 9.4	2.1 × 10^−10^
Body mass index (kg/m^2^)	612	24.3 ± 3.5	23.4 ± 3.3	24.1 ± 3.2	25.6 ± 3.4	4.1 × 10^−10^
Diabetes mellitus (%)	612	5.1	5.7	5.7	4.6	0.643
Current smokers (%)	612	19.3	15.5	18.7	25.8	0.012
Obesity (%)	612	39.5	28	33.7	58.8	4.1 × 10^−10^
Hypertension (%)	612	19.4	12.4	15.5	30.4	1.1 × 10^−5^
Insulin resistance (%)	612	24.8	16.1	23.3	36.1	2.6 × 10^−5^
Metabolic syndrome (%)	612	18.5	11.9	14	31.4	5.6 × 10^−7^
Systolic BP^∗^ (mmHg)	546	113.1 ± 16.1	109.3 ± 14.8	113.8 ± 16.2	117.5 ± 16.1	9.0 × 10^−6^
Diastolic BP^∗^ (mmHg)	546	75.0 ± 10.0	73.4 ± 9.6	75.2 ± 8.9	77.0 ± 10.7	3.6 × 10^−3^
Mean BP^∗^ (mmHg)	546	87.7 ± 11.2	85.3 ± 10.6	88.1 ± 10.7	90.5 ± 11.4	8.6× 10^−5^
Total cholesterol^#^ (mg/dL)	603	198.8 ± 36.4	194.9 ± 34.9	200.4 ± 39.6	202.5 ± 34.3	0.097
HDL-C^#^ (mg/dL)	603	53.0 (45.0–65.0)	55.0 (46.0–67.0)	54.0 (47.0–63.8)	49.0 (43.0–59.0)	0.002
LDL-C^#^ (mg/dL)	603	116.1 ± 32.9	113.3 ± 31.7	118.2 ± 36.0	117.6 ± 30.7	0.309
Triglyceride^#^ (mg/dL)	603	115.0 (76.0–165.8)	92.5 (62.3–142.0)	106.5 (80.3–157.0)	143.0 (104.0–197.0)	7.8 × 10^−10^
Fasting plasma glucose^∗∗^ (mg/dL)	601	92.0 (88.0–98.0)	92.0 (87.8–97.3)	93.0 (88.0–99.0)	94.0 (88.0–99.0)	0.513
Fasting serum insulin^∗∗^ (*μ*U/mL)	601	8.0 (6.1–10.9)	6.8 (5.5–9.2)	7.8 (6.0–10.9)	9.6 (7.3–12.7)	1.4 × 10^−12^
HOMA-IR index^∗∗^	601	1.9 (1.4–2.6)	1.6 (1.2–2.2)	1.8 (1.4–2.5)	2.3 (1.7–2.9)	5.8 × 10^−10^
Creatinine (mg/dL)	511	0.99 ± 0.46	0.95 ± 0.18	0.96 ± 0.19	1.07 ± 0.76	0.03
eGFR (mL/min/1.86 m^2^)	511	83.2 ± 14.2	83.2 ± 14.2	82.1 ± 12.9	76.7 ± 16.0	9.6 × 10^−5^
Chemerin (ng/mL)	580	125.8 (101.0–148.0)	93.4 (82.5–101.0)	125.5 (116.7–132.6)	164.3 (147.6–185.8)	1.8 × 10^−167^

Data are presented as means ± SD, percentage, or median (interquartile range) as appropriate. BP: blood pressure; HDL: high-density lipoprotein; LDL: low-density lipoprotein; HOMA-IR index: homeostasis model assessment of insulin resistance index; eGFR: estimated glomerular filtration rate. ^∗^546 were analyzed with the exclusion of subjects using antihypertensive drugs. ^#^603 were analyzed with the exclusion of subjects using lipid-lowering agents. ^∗∗^601 were analyzed with the exclusion of subjects using hypoglycemic agent.

**Table 2:** Association between circulating chemerin levels and measurable cardiovascular risk factors in the Taiwanese population.

Clinical and biochemical parameters		Unadjusted		Adjusted for age and sex			Adjusted for age, sex, and smoking		
		*r*	*P* value	*r*	*P* value	Adjusted *P* value	*r*	*P* value	Adjusted *P* value
Anthropology	Age (years)	0.157	1.41 × 10^−4^	—	—	—	—	—	—
	Waist circumference (cm)	0.257	3.18 × 10^−10^	0.259	2.78 × 10^−10^	<0.001	0.254	6.21 × 10^−10^	<0.001
	Body mass index (kg/m^2^)	0.259	2.42 × 10^−10^	0.261	1.73 × 10^−10^	<0.001	0.264	1.11 × 10^−10^	<0.001
Blood pressure	Systolic BP (mmHg)	0.189	1.60 × 10^−5^	0.144	0.001	0.017	0.147	0.001	0.017
	Diastolic BP (mmHg)	0.158	0.000319	0.136	0.002	0.034	0.145	0.001	0.017
	Mean BP (mmHg)	0.185	2.30 × 10^−5^	0.150	0.001	0.017	0,157	3.56 × 10^−4^	<0.005
Lipid profiles	Total cholesterol (mg/dL)	0.067	0.111	0.041	0.333	1.0	0.035	0.402	1.0
	HDL-C (mg/dL)	−0.143	0.001	−0.176	4.50 × 10^−5^	<0.001	−0.159	1.45 × 10^−4^	<0.002
	LDL-C (mg/dL)	0.041	0.331	0.017	0.691	1.0	0.023	0.587	1.0
	Triglyceride (mg/dL)	0.252	1.03 × 10^−9^	0.263	2.00 × 10^−10^	<0.001	0.241	5.57 × 10^−9^	<0.001
Glucose metabolism	Fasting plasma glucose (mg/dL)	−0.017	0.686	−0.026	0.532	1.0	−0.028	0.504	1.0
	Serum insulin (*μ*U/mL)	0.274	3.14 × 10^−11^	0.289	2.33 × 10^−12^	<0.001	0.283	6.47 × 10^−12^	<0.001
	HOMA-IR index	0.240	6.46 × 10^−9^	0.252	1.14 × 10^−9^	<0.001	0.247	2.78 × 10^−9^	<0.001
Renal function	Creatinine (mg/dL)	0.290	1.10 × 10^−10^	0.299	2.59 × 10^−11^	<0.001	0.306	8.02 × 10^−12^	<0.001
	eGFR (mL/min/1.86 m^2^)	−0.250	2.91 × 10^−8^	−0.196	1.60 × 10^−5^	<0.001	−0.202	8.0 × 10^−6^	<0.001

Abbreviations as in Table 1. For Bonferroni correction, *n* = 15.

**Table 3:** Chemerin levels according to the cardiovascular risk factors.

		*N*	Chemerin levels Means ± SD	*P* value	Adjusted *P* value
Sex	Male	317	129.2 ± 42.2	0.044	0.231
	Female	263	131.1 ± 46.0		
Current smoker	No	464	128.4 ± 44.5	0.009	0.063
	Yes	116	137.0 ± 41.0		
Hypertension	No	467	125.9 ± 38.0	0.015	0.105
	Yes	113	147.5 ± 60.0		
Diabetes mellitus	No	549	130.1 ± 44.2	0.380	1.0
	Yes	31	129.3 ± 40.6		
Obesity	No	347	121.1 ± 36.9	6.52 × 10^−9^	4.55 × 10^−8^
	Yes	233	143.4 ± 50.0		
Insulin resistance	No	434	126.5 ± 44.3	0.001	0.007
	Yes	146	140.7 ± 41.4		
Metabolic syndrome	No	469	125.6 ± 40.1	0.004	0.021
	Yes	111	148.9 ± 53.6		

SD: standard deviation; *N*: number. *P* value: adjusted for age and sex, body mass index (BMI), and current smoker. Sex: adjusted for age, BMI, and current smoker. Current smoker: adjusted for age, sex, and BMI. Obesity: adjusted for age, sex, and current smoker. For Bonferroni correction, *n* = 7.

**Table 4:** Association between circulating chemerin levels and inflammatory marker and adipokine levels.

Clinical biochemical parameters		Unadjusted		Adjusted for age and sex			Adjusted for age, sex, and smoking		
		*r*	*P* value	*r*	*P* value	Adjusted *P* value	*r*	*P* value	Adjusted *P* value
Inflammatory markers	CRP (mg/L)	0.361	2.50 × 10^−19^	0.349	5.26 × 10^−18^	<0.001	0.339	5.92 × 10^−17^	<0.001
	Fibrinogen (*μ*mol/L)	0.165	6.22 × 10^−5^	0.137	0.001	0.015	0.132	0.001	0.015
	SAA (*μ*g/mL)	0.245	3.41 × 10^−9^	0.233	2.05 × 10^−8^	<0.001	0.232	2.65 × 10^−8^	<0.001
	sE-Selectin (g/L)	0.134	0.001	0.146	4.69 × 10^−4^	0.007	0.132	0.002	0.030
	sP-Selectin (ng/mL)	0.129	0.002	0.128	0.002	0.030	0.123	0.003	0.045
	sVCAM1 (g/L)	−0.028	0.498	−0.056	0.178	1.0	−0.059	0.159	1.0
	sICAM1 (g/L)	0.122	0.003	0.114	0.006	1.0	0.094	0.024	1.0
	MMP1 (pg/mL)	0.113	0.007	0.121	0.004	1.0	0.113	0.006	1.0
	sTNFRII (pg/mL)	0.141	0.001	0.123	0.003	0.045	0.119	0.004	1.0
	MMP2 (ng/mL)	−0.104	0.012	−0.13	0.002	0.030	−0.120	0.004	1.0
	MMP9 (mg/L)	0.063	0.136	0.08	0.058	1.0	0.061	0.149	1.0
	MCP1 (pg/mL)	0.045	0.282	0.043	0.303	1.0	0.040	0.335	1.0
	IL6 (pg/L)	0.139	0.001	0.137	0.001	0.015	0.131	0.002	0.030
Adipokines	Adiponectin (mg/L)	−0.128	0.002	−0.165	6.8 × 10^−5^	0.001	−0.158	1.42 × 10^−4^	0.002
	Leptin (g/L)	0.299	1.89 × 10^−13^	0.36	4.21 × 10^−19^	<0.001	0.359	4.95 × 10^−19^	<0.001
	Resistin (ng/mL)	0.051	0.234	0.047	0.274	1.0	0.041	0.330	1.0
	Lipocalin 2 (ng/mL)	0.159	1.66 × 10^−4^	0.165	9.2 × 10^−5^	0.001	0.161	1.37 × 10^−4^	0.002
	GDF15 (pg/mL)	0.167	5.20 × 10^−5^	0.109	0.009	1.0	0.091	0.027	1.0

Data are presented as means ± SD or median (interquartile range) as appropriate. CRP: C-reactive protein; SAA: serum amyloid A; sE-selectin: soluble E-selectin; sP-selectin: soluble P-selectin; sVCAM1: soluble vascular cell adhesive molecule 1; sICAM1: soluble intercellular adhesive molecule 1; sTNFRII: soluble tumor necrosis factor-alpha receptor 2; MMP1: matrix metalloproteinase 1; MMP2: matrix metalloproteinase 2; MMP9: matrix metalloproteinase 9; MCP1: monocyte chemotactic protein 1; IL6: interleukin 6; GDF15: growth differentiation factor 15. For Bonferroni correction, *n* = 18.

**Table 5:** Association of *RARRES2* gene polymorphisms with chemerin level.

SNP number	Gene	Position	MAF (MA)	MM	Mm	mm	*β*(SE)	*P* value	MM	Mm + mm	*P* value
rs7806429	*RARRES2*	3′UTR	0.410 (C)	125.38 ± 47.13 (196)	130.60 ± 41.40 (285)	140.87 ± 43.42 (90)	0.027 (0.008)	0.001	125.38 ± 47.13 (196)	133.07 ± 42.07 (375)	0.009
rs4721	*RARRES2*	Intron	0.476 (C)	124.99 ± 48.87 (156)	130.18 ± 41.26 (289)	138.36 ± 43.02 (124)	0.023 (0.008)	0.002	124.99 ± 48.87 (156)	132.64 ± 41.91 (413)	0.019
rs17173608	*RARRES2*	Intron	0.067 (G)	130.72 ± 44.24 (499)	127.77 ± 41.41 (68)	151.39 ± 54.85 (4)	0.002 (0.015)	0.912	130.72 ± 44.24 (499)	129.08 ± 42.13 (72)	0.901
rs3735167	*RARRES2*	5′UTR	0.284 (T)	125.11 ± 45.26 (292)	133.79 ± 47.77 (231)	147.16 ± 36.54 (46)	0.038 (0.008)	5.27 × 10^−6^	125.11 ± 45.26 (292)	136.16 ± 40.27 (277)	4.33 × 10^−5^
rs10244748	*RARRES2*	5′UTR	0.214 (C)	130.06 ± 37.82 (351)	132.53 ± 53.33 (194)	121.26 ± 46.48 (23)	−0.013 (0.009)	0.174	130.06 ± 37.82 (351)	131.34 ± 52.67 (217)	0.353
rs10282458	*RARRES2*	5′UTR	0.283 (A)	125.61 ± 45.79 (289)	133.59 ± 42.42 (236)	147.02 ± 35.37 (43)	0.038 (0.008)	1.08 × 10^−5^	125.61 ± 45.79 (289)	135.66 ± 41.64 (279)	8.91 × 10^−5^

*P* value adjusted for age, sex, body mass index, and smoking, and antihypertensive, antidiabetic, and lipid-lowering drugs. MAF: minor allele frequency; MA: minor allele; MM: homozygosity of major allele; Mm: heterozygosity; mm: homozygosity of minor allele.

**Table 6:** Haplotype analysis.

	Haplotype	Frequency	Chemerin level		Fibrinogen level		MMP9 level		IL6 level		LCN2 level		CRP level	
			Coefficient	*P* value	Coefficient	*P* value	Coefficient	*P* value	Coefficient	*P* value	Coefficient	*P* value	Coefficient	*P* value
H1	TTTCTG	47.94%	−0.041	0.008	−0.022	0.116	−0.051	0.091	−0.106	0.036	−0.032	0.210	−0.063	0.292
H2	CGTTTA	25.75%	0.065	0.0002	0.042	0.007	0.064	0.059	0.133	0.016	0.071	0.011	0.130	0.053
H3	CGTCCG	11.32%	−0.024	0.331	0.004	0.849	0.015	0.750	−0.049	0.531	−0.016	0.684	−0.060	0.521
H4	TGGCCG	5.17%	−0.040	0.238	−0.059	0.048	0.073	0.257	0.034	0.751	0.038	0.474	−0.052	0.687
H5	TTTCCG	3.71%	−0.018	0.677	−0.015	0.687	−0.104	0.208	0.040	0.776	−0.026	0.708	−0.059	0.718
H6	CGTCTG	1.85%	−0.041	0.482	−0.062	0.230	−0.199	0.083	−0.024	0.901	−0.135	0.154	0.021	0.926

*P* value adjusted for age, sex, body mass index, and smoking, and antihypertensive, antidiabetic, and lipid-lowering drugs. Abbreviations as in Table 2. The haplotype alleles correspond to rs7806429, rs4721, rs17173608, rs3735167, rs10244748, and rs10282458.

**Table 7:** Chemerin levels: stepwise linear regression analysis, including genotypes.

	Beta	*R* ^2∗^	*P* value
CRP level	0.053	0.103	<0.001
Leptin level	0.125	0.154	<0.001
Serum triglyceride level	0.077	0.182	0.002
eGFR	−0.001	0.215	<0.001
*RARRES2* rs3735167 CC genotype	0.036	0.232	0.001
Sex (male versus female)	−0.040	0.248	0.012
sP-Selectin level	0.044	0.258	0.019

^∗^Cumulative *R* ^2^. Abbreviations as in Tables 1 and 3.

## Supplementary Materials

Supplementary Materials Supplementary Table 1: the biomarker intra- and interassay variability of coefficients were measured, and all were within the range of 1.8% to 10.9%. Supplementary Table 2: circulating inflammatory markers and adipokine levels were analyzed according to tertiles of circulating chemerin levels. Supplementary Figure 1: linkage disequilibrium of the *RARRES2* polymorphisms was analyzed. Strong linkage disequilibrium was observed between SNPs rs7806429 and rs4721 and between SNPs rs3735167 and rs10282458.

## References

[1] Park S. E., Park C. Y., Sweeney G. (2015). Biomarkers of insulin sensitivity and insulin resistance: past, present and future. *Critical Reviews in Clinical Laboratory Sciences*.

[2] Bozaoglu K., Bolton K., McMillan J. (2007). Chemerin is a novel adipokine associated with obesity and metabolic syndrome. *Endocrinology*.

[3] Hart R., Greaves D. R. (2010). Chemerin contributes to inflammation by promoting macrophage adhesion to VCAM-1 and fibronectin through clustering of VLA-4 and VLA-5. *The Journal of Immunology*.

[4] Goralski K. B., McCarthy T. C., Hanniman E. A. (2007). Chemerin, a novel adipokine that regulates adipogenesis and adipocyte metabolism. *Journal of Biological Chemistry*.

[5] Sell H., Divoux A., Poitou C. (2010). Chemerin correlates with markers for fatty liver in morbidly obese patients and strongly decreases after weight loss induced by bariatric surgery. *The Journal of Clinical Endocrinology and Metabolism*.

[6] Zabel B. A., Allen S. J., Kulig P. (2005). Chemerin activation by serine proteases of the coagulation, fibrinolytic, and inflammatory cascades. *Journal of Biological Chemistry*.

[7] Weigert J., Neumeier M., Wanninger J. (2010). Systemic chemerin is related to inflammation rather than obesity in type 2 diabetes. *Clinical Endocrinology*.

[8] Lehrke M., Becker A., Greif M. (2009). Chemerin is associated with markers of inflammation and components of the metabolic syndrome but does not predict coronary atherosclerosis. *European Journal of Endocrinology*.

[9] Neves K. B., Nguyen Dinh Cat A., Lopes R. A. (2015). Chemerin regulates crosstalk between adipocytes and vascular cells through Nox. *Hypertension*.

[10] Roman A. A., Parlee S. D., Sinal C. J. (2012). Chemerin: a potential endocrine link between obesity and type 2 diabetes. *Endocrine*.

[11] Bozaoglu K., Curran J. E., Stocker C. J. (2010). Chemerin, a novel adipokine in the regulation of angiogenesis. *The Journal of Clinical Endocrinology & Metabolism*.

[12] Kaur J., Adya R., Tan B. K., Chen J., Randeva H. S. (2010). Identification of chemerin receptor (ChemR23) in human endothelial cells: chemerin-induced endothelial angiogenesis. *Biochemical and Biophysical Research Communications*.

[13] Rourke J. L., Dranse H. J., Sinal C. J. (2013). Towards an integrative approach to understanding the role of chemerin in human health and disease. *Obesity Reviews*.

[14] Kaneko K., Miyabe Y., Takayasu A. (2011). Chemerin activates fibroblast-like synoviocytes in patients with rheumatoid arthritis. *Arthritis Research & Therapy*.

[15] Weigert J., Obermeier F., Neumeier M. (2010). Circulating levels of chemerin and adiponectin are higher in ulcerative colitis and chemerin is elevated in Crohn's disease. *Inflammatory Bowel Diseases*.

[16] Adrych K., Stojek M., Smoczynski M., Sledzinski T., Sylwia S. W., Swierczynski J. (2012). Increased serum chemerin concentration in patients with chronic pancreatitis. *Digestive and Liver Disease*.

[17] Pfau D., Bachmann A., Lössner U. (2010). Serum levels of the adipokine chemerin in relation to renal function. *Diabetes Care*.

[18] Kukla M., Zwirska-Korczala K., Hartleb M. (2010). Serum chemerin and vaspin in non-alcoholic fatty liver disease. *Scandinavian Journal of Gastroenterology*.

[19] Mariani F., Roncucci L. (2015). Chemerin/chemR23 axis in inflammation onset and resolution. *Inflammation Research*.

[20] Watts S. W., Dorrance A. M., Penfold M. E. (2013). Chemerin connects fat to arterial contraction. *Arteriosclerosis, Thrombosis, and Vascular Biology*.

[21] Yan Q., Zhang Y., Hong J. (2012). The association of serum chemerin level with risk of coronary artery disease in Chinese adults. *Endocrine*.

[22] Zhao D., Bi G., Feng J., Huang R., Chen X. (2015). Association of serum chemerin levels with acute ischemic stroke and carotid artery atherosclerosis in a Chinese population. *Medical Science Monitor*.

[23] Tönjes A., Scholz M., Breitfeld J. (2014). Genome wide meta-analysis highlights the role of genetic variation in *RARRES2* in the regulation of circulating serum chemerin. *PLoS Genetics*.

[24] Hashemi M., Rezaei H., Eskandari-Nasab E., Kaykhaei M. A., Zakeri Z., Taheri M. (2012). Association between *chemerin rs17173608* and *vaspin rs2236242* gene polymorphisms and the metabolic syndrome, a preliminary report. *Gene*.

[25] Min J. L., Nicholson G., Halgrimsdottir I. (2012). Coexpression network analysis in abdominal and gluteal adipose tissue reveals regulatory genetic loci for metabolic syndrome and related phenotypes. *PLoS Genetics*.

[26] Mussig K., Staiger H., Machicao F. (2009). *RARRES2*, encoding the novel adipokine chemerin, is a genetic determinant of disproportionate regional body fat distribution: a comparative magnetic resonance imaging study. *Metabolism Clinical and Experimental*.

[27] expert consultation W. H. O. (2004). Appropriate body-mass index for Asian populations and its implications for policy and intervention strategies. *The Lancet*.

[28] Ko Y. L., Hsu L. A., Hsu K. H., Ko Y. H., Lee Y. S. (2004). The interactive effects of hepatic lipase gene promoter polymorphisms with sex and obesity on high-density-lipoprotein cholesterol levels in Taiwanese-Chinese. *Atherosclerosis*.

[29] Hsu L. A., Ko Y. L., Wu S. (2009). Association of soluble intercellular adhesion molecule-1 with insulin resistance and metabolic syndrome in Taiwanese. *Metabolism Clinical and Experimental*.

[30] Matsuo S., Imai E., Horio M. (2009). Revised equations for estimated GFR from serum creatinine in Japan. *American Journal of Kidney Diseases*.

[31] Li Y., Shi B., Li S. (2014). Association between serum chemerin concentrations and clinical indices in obesity or metabolic syndrome: a meta-analysis. *PLoS One*.

[32] Aydın K., Canpolat U., Akın Ş. (2016). Chemerin is not associated with subclinical atherosclerosis markers in prediabetes and diabetes. *The Anatolian Journal of Cardiology*.

[33] Yang M., Yang G., Dong J. (2010). Elevated plasma levels of chemerin in newly diagnosed type 2 diabetes mellitus with hypertension. *Journal of Investigative Medicine*.

[34] Jung U. J., Choi M. S. (2014). Obesity and Its metabolic complications: the role of adipokines and the relationship between obesity, inflammation, insulin resistance, dyslipidemia and nonalcoholic fatty liver disease. *International Journal of Molecular Sciences*.

[35] Sell H., Laurencikiene J., Taube A. (2009). Chemerin is a novel adipocyte-derived factor inducing insulin resistance in primary human skeletal muscle cells. *Diabetes*.

[36] Ouwens D. M., Bekaert M., Lapauw B. (2012). Chemerin as biomarker for insulin sensitivity in males without typical characteristics of metabolic syndrome. *Archives of Physiology and Biochemistry*.

[37] Leiherer A., Muendlein A., Kinz E. (2016). High plasma chemerin is associated with renal dysfunction and predictive for cardiovascular events—insights from phenotype and genotype characterization. *Vascular Pharmacology*.

[38] Zylla S., Pietzner M., Kühn J. P. (2017). Serum chemerin is associated with inflammatory and metabolic parameters—results of a population-based study. *Obesity*.

[39] Landgraf K., Friebe D., Ullrich T. (2012). Chemerin as a mediator between obesity and vascular inflammation in children. *The Journal of Clinical Endocrinology & Metabolism*.

[40] Wang C., Wu W. K., Liu X. (2014). Increased serum chemerin level promotes cellular invasiveness in gastric cancer: a clinical and experimental study. *Peptides*.

[41] Bozaoglu K., Segal D., Shields K. A. (2009). Chemerin is associated with metabolic syndrome phenotypes in a Mexican-American population. *The Journal of Clinical Endocrinology & Metabolism*.

[42] Chu S. H., Lee M. K., Ahn K. Y. (2012). Chemerin and adiponectin contribute reciprocally to metabolic syndrome. *PLoS One*.

[43] Hsu L. A., Wu S., Juang J. J. (2017). Growth differentiation factor 15 may predict mortality of peripheral and coronary artery diseases and correlate with their risk factors. *Mediators of Inflammation*.

[44] Wu S., Hsu L. A., Teng M. S. (2010). Association of matrix metalloproteinase 9 genotypes and cardiovascular disease risk factors with serum matrix metalloproteinase 9 concentrations in Taiwanese individuals. *Clinical Chemistry and Laboratory Medicine*.

[45] Wu S., Hsu L. A., Cheng S. T. (2014). Circulating YKL-40 level, but not *CHI3L1* gene variants, is associated with atherosclerosis-related quantitative traits and the risk of peripheral artery disease. *International Journal of Molecular Sciences*.

[46] Teng M. S., Hsu L. A., Wu S., Sun Y. C., Juan S. H., Ko Y. L. (2015). Association of *CDH13* genotypes/haplotypes with circulating adiponectin levels, metabolic syndrome, and related metabolic phenotypes: the role of the suppression effect. *PLoS One*.

[47] Teng M. S., Wu S., Hsu L. A., Chou H. H., Ko Y. L. (2015). Differential associations between *CDH13* genotypes, adiponectin levels, and circulating levels of cellular adhesive molecules. *Mediators of Inflammation*.

[48] Ko Y. L., Hsu L. A., Wu S., Teng M. S., Chou H. H. (2016). CRP and SAA1 haplotypes are associated with both C-reactive protein and serum amyloid a levels: role of suppression effects. *Mediators of Inflammation*.

[49] Thomas M. R., Lip G. Y. (2017). Novel risk markers and risk assessments for cardiovascular disease. *Circulation Research*.

[50] Mehanna E. T., Mesbah N. M., Ghattas M. H., Saleh S. M., Abo-Elmatty D. M. (2016). Association of chemerin rs17173608 and vaspin rs2236242 gene polymorphisms with metabolic syndrome in Egyptian women. *Endocrine Research*.

[51] Movahed Z., Kohan L., Fallahi S., Tabiee O. (2015). Influence of *chemerin* rs17173608 polymorphism on polycystic ovary syndrome susceptibility. *Taiwanese Journal of Obstetrics & Gynecology*.

[52] Huang H. W., Liang B. Y., Li Y. X. (2016). Association of Polymorphisms in STRA6 and RARRES2 genes with type 2 diabetes in Southern Han Chinese. *BioMed Research International*.

[53] Zhao L., Yamaguchi Y., Sharif S. (2011). Chemerin158K protein is the dominant chemerin isoform in synovial and cerebrospinal fluids but not in plasma. *Journal of Biological Chemistry*.

