# Supplementary material for: Pleiotropic Associations of RARRES2 Gene Variants and Circulating Chemerin Levels: Potential Roles of Chemerin Involved in the Metabolic and Inflammation-Related Diseases
Source: Mediators Inflamm. 2018 Mar 12;2018:4670521. doi: 10.1155/2018/4670521 (PMC5867667; doi:10.1155/2018/4670521)

**Supplementary References:**

1. Chang PY, Wu TL, Tsao KC, et al. Microplate ELISAs for soluble VCAM-1 and ICAM-1. Ann Clin Lab Sci 2005; 35:312-7.

2. Tsao KC, Chang PY, Li CC, Wu TL, Sun CF, Wu JT. Development of a microplate ELISA for circulating E-selectin: assay characterization, comparison with a commercial kit, wand establishment of normal reference values. J Clin Lab Anal 2003; 17:97-101.

3. Wu TL, I Chen Tsai, Chang PY, et al. Establishment of an in-house ELISA and the reference range for serum amyloid A (SAA): complementarity between SAA and C-reactive protein as markers of inflammation. Clin Chim Acta 2007; 376:72-6.

4. Wu TL, Tsao KC, Chang CP, Li CN, Sun CF, Wu JT. Development of ELISA on microplate for serum C-reactive protein and establishment of age-dependent normal reference range. Clin Chim Acta 2002; 322:163-8.

Supplementary Table 1. Biomarker inter- and intra-assay variability measures

| Biomarker | Source | Intra-assay | Inter-assay |
| --- | --- | --- | --- |
| Total cholesterol# (mg/dL) | Serum | CV = 2.8% | CV = 2.8% |
| HDL-cholesterol# (mg/dL) | Serum | CV = 4.0% | CV = 4.1% |
| LDL-cholesterol# (mg/dL) | Serum | CV = 2.2% | CV = 2.8% |
| Triglyceride# (mg/dL ) | Serum | CV = 2.1% | CV = 3.4% |
| Fasting plasma glucose** (mg/dl) | Plasma | CV = 1.2% | CV = 1.4% |
| Fasting serum insulin** (μU/ml) | serum | CV = 8.1% | CV = 8.2% |
| Creatinine (mg/dL) | serum | CV = 1.4% | CV = 1.9% |
| Chemerin (ng/mL) | plasma | CV = 1.4% | CV = 4.2% |
| CRP (mg/L) | Serum | CV = 7.1% | CV = 10.9% |
| Fibrinogen (μmol/L) | Plasma | CV = 3.9% | CV = 5.3% |
| SAA (μg/mL) | serum | CV = 8.5% | CV = 8.1% |
| sE-selectin(g/L) | serum | CV = 6.5% | CV = 3.4% |
| sP- selectin(ng/mL) | plasma | CV = 0.6% | CV = 2.9% |
| sVCAM1(g/L) | serum | CV = 5.2% | CV = 6.8% |
| sICAM1 (g/L) | serum | CV = 2.1% | CV = 3.3% |
| sTNFRII (pg/mL) | plasma | CV = 1.4% | CV =4.7% |
| MMP1 (pg/mL) | plasma | CV = 5.7% | CV = 8.2% |
| MMP2 (ng/mL) | serum | CV = 6.1% | CV = 7.1% |
| MMP9 (mg/L) | serum | CV = 7.1% | CV = 9.1% |
| MCP1 (pg/mL) | plasma | CV = 2.4% | CV = 3.6% |
| IL6 (pg/L) | serum | CV = 5.7% | CV = 8.0% |
| Adiponectin (mg/L) | serum | CV = 4.0% | CV = 3.7% |
| Leptin (g/L) | serum | CV = 5.3% | CV = 6.0% |
| Resistin (ng/mL) | serum | CV = 3.4% | CV = 4.0% |
| Lipocalin 2 (ng/mL) | serum | CV = 1.4% | CV= 5.2% |
| GDF15 (pg/mL) | plasma | CV = 1.2% | CV= 5.5% |

Abbreviations as in Table 1 and Supplementary Table 2

CV: coefficient of variation

Supplementary Table 2. Circulating inflammatory marker and adipokine levels according to tertiles of circulating chemerin levels

|  |  | N | Total | 1st tertile | 2nd tertile | 3rd tertile | p-Value |
| --- | --- | --- | --- | --- | --- | --- | --- |
| Inflammatory markers | CRP (mg/L) | 612 | 0.63 (0.27-1.34) | 0.38 (0.19-0.78) | 0.57 (0.24-1.15) | 1.1 (0.54-2.52) | 3.2 × 10^-20^ |
|  | Fibrinogen (μmol/L) | 612 | 264.9 ± 70.3 | 250.2 ± 63.2 | 258.6 ± 67.5 | 282.8 ± 74.0 | 2.9 × 10^-5^ |
|  | SAA (μg/mL) | 599 | 3.6 (1.7-6.2) | 2.3 (1.2-5.0) | 3.7 (1.7-6.0) | 4.4 (2.6-7.3) | 2.0 × 10^-6^ |
|  | sE-selectin(g/L) | 605 | 50.4 (36.0-65.9) | 46.2 (34.6-58.9) | 51.0 (37.3-66.4) | 54.9 (37.5-76.0) | 0.002 |
|  | sP- selectin(ng/mL) | 610 | 94.7 (65.7-169.2) | 85.2 (63.6-130.0) | 100.4 (66.7-186.4) | 106.8 (70.1-223.4) | 1.4 × 10^-4^ |
|  | sVCAM1(g/L) | 607 | 479.0 (409.0-549.0) | 480.0 (409.5-553.8) | 474.5 (405.8-546.0) | 476.0 (405.5-551.5) | 0.77 |
|  | sICAM1 (g/L) | 606 | 231.1 (180.8-278.2) | 204.7 (168.0-252.9) | 236.5 (191.5-283.3) | 251.8 (201.8-299.2) | 0.002 |
|  | sTNFRII (pg/mL) | 610 | 3107.3 (2653.6-3740.0) | 2990.5 (2614.9-3583.7) | 3073.1 (2634.6-3674.1) | 3213.1 (2756.5-3821.7) | 0.002 |
|  | MMP1 (pg/mL) | 610 | 188.1 (100.4-399.1) | 153.8 (85.2-317.3) | 198.7 (110.4-454.8) | 211.8 (115.4-465.6) | 0.005 |
|  | MMP2 (ng/mL) | 607 | 121.9 (102.7-140.1) | 123.0 (107.0-140.9) | 122.3 (100.3-142.7) | 114.7 (94.8-135.2) | 0.016 |
|  | MMP9 (mg/L) | 597 | 112.5 (75.6-169.1) | 117.8 (73.1-180.4) | 106.4 (74.3-162.8) | 117.6 (85.9-175.4) | 0.27 |
|  | MCP1 (pg/mL) | 610 | 59.8 (42.7-82.7) | 57.1 (37.9-83.3) | 60.3 (43.8-81.9) | 61.5 (47.3-83.1) | 0.59 |
|  | IL6 (pg/L) | 587 | 2.3 (1.3-4.2) | 1.8 (1.0-3.5) | 2.2 (1.2-4.1) | 2.6 (1.6-4.7) | 2.0 × 10^-4^ |
| Adipokines | Adiponectin (mg/L) | 612 | 6.0 (3.7-9.2) | 6.9 (3.9-10.3) | 5.7 (3.8-8.3) | 5.3 (3.2-7.8) | 0.001 |
|  | Leptin (g/L) | 612 | 14.90 (8.15-25.95) | 10.2 (5.6-18.3) | 14.0 (7.4-25.7) | 20.5 (12.2-31.1) | 8.1 × 10^-15^ |
|  | Resistin (ng/mL) | 587 | 14.8 (10.3-22.6) | 14.7 (10.2-23.2) | 14.6 (10.0-21.0) | 15.3 (10.8-23.3) | 0.22 |
|  | Lipocalin2 (ng/mL) | 587 | 71.8 (53.2-94.1) | 69.4 (51.8-89.4) | 72.7 (51.0-95.6) | 74.3 (55.9-97.7) | 0.096 |
|  | GDF15 (pg/mL) | 592 | 535.0 (415.3-716.5) | 482.0 (375.5-616.0) | 549.0 (420.0-702.5) | 594 (448.5-867.8) | 7.2 × 10^-5^ |

Data are presented as mean ± SD or median (inter-quartile range) as appropriate. CRP, C-reactive protein; SAA, serum amyloid A; sE-selectin, soluble E-selectin; sP-selectin, soluble P-selectin;sVCAM1, soluble vascular cell adhesive molecule 1; sICAM1, soluble intercellular adhesive molecule 1; sTNFRII, soluble tumor necrosis factor-alpha receptor 2; MMP1, matrix metalloproteinase 1; MMP2, matrix metalloproteinase 2; MMP9, matrix metalloproteinase 9; MCP1, monocyte chemotactic protein 1; GDF15, growth differentiation factor 15

Supplementary Figure 1: Linkage disequilibrium of the *RARRES2* polymorphisms


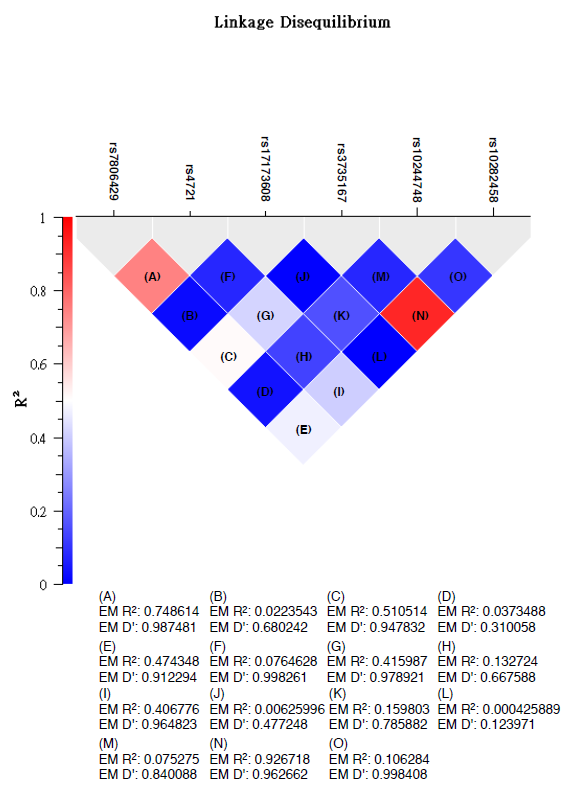

Supplement: Supplementary Materials — Supplementary Table 1: the biomarker intra- and interassay variability of coefficients were measured, and all were within the range of 1.8% to 10.9%. Supplementary Table 2: circulating inflammatory markers and adipokine levels were analyzed according to tertiles of circulating chemerin levels. Supplementary Figure 1: linkage disequilibrium of the RARRES2 polymorphisms was analyzed. Strong linkage disequilibrium was observed between SNPs rs7806429 and rs4721 and between SNPs rs3735167 and rs10282458. [file 4670521.f1.doc]
